# Reading fluency as a measure of educational level

**DOI:** 10.1590/1980-57642021dn15-030008

**Published:** 2021

**Authors:** Alberto Luis Fernandez, Gabriel Jauregui Arriondo

**Affiliations:** 1 Universidad Católica de Córdoba – Córdoba, Argentina.

**Keywords:** educational measurement, reading, cognitive function, neuropsychological tests, avaliação educacional, leitura, cognição, testes neuropsicológicos

## Abstract

Education exerts a powerful influence on the performance on neuropsychological tests. Recently, the number of years that a person attends school has been the preferred method to operationalize educational level (EL). However, reading fluency (RF) has emerged as an alternative method that can define the quality of education.

**Objective:**

To compare the influence of the number of years of education with RF on the cognitive performance in a control sample.

**Methods:**

Fifty-six control participants with varying ages (17–87 years) and levels of education (3–19 years of formal schooling) were administered a neuropsychological scale along with an RF task. This scale measured attention, memory, language, executive functions, and constructional praxis. The RF task consisted of a short text. The score was the number of words read correctly per minute. Pearson’s *r* was used to compute correlations.

**Results:**

Results showed that RF had a higher correlation (0.53) than the years of schooling (0.38) with the scores of the neuropsychological scale.

**Conclusions:**

Reading fluency is a short, practical task that is easy to use in different languages and is a promising tool for EL assessment. It is also an adequate alternative to the reading of irregular words as a qualitative measure of EL.

## RESUMO

A educação exerce uma poderosa influência no desempenho em testes neuropsicológicos. Até o momento, o número de anos que uma pessoa frequenta a escola tem sido o método preferido para operacionalizar o nível educacional (NE). No entanto, a fluência de leitura (FL) surge como um método alternativo que pode informar a qualidade do ensino.

**Objetivo:**

comparar a influência do número de anos de estudo *versus* FL no desempenho cognitivo em uma amostra controle.

**Métodos:**

Administrou-se uma escala neuropsicológica juntamente com uma tarefa de FL a 56 participantes-controle com idades variáveis (17–87) e diferentes níveis de educação (3–19 anos de escolaridade formal). A escala mediu atenção, memória, linguagem, funções executivas e práxis construtiva. A tarefa de FL consistiu em um texto curto. A pontuação foi o número de palavras lidas corretamente por minuto. O r de Pearson foi usado para calcular correlações.

**Resultados:**

Os resultados mostraram que a FL tinha uma correlação mais elevada (0,53) do que anos de escola (0,38) com as pontuações da escala neuropsicológica.

**Conclusões:**

A fluência de leitura é uma tarefa prática, curta e de fácil aplicação em diferentes idiomas, que se destaca como uma ferramenta promissora para a avaliação do NE. Também é uma alternativa adequada à leitura de palavras irregulares como uma medida qualitativa de NE.

## INTRODUCTION

Neuropsychological performance is highly influenced by culture. Culture affects the expression of the basic cognitive functions.^1^ Therefore, studying the influence of the elements ofculture on cognitive performance has become highly relevant in modern neuropsychology. One of the most significant elements of culture is education. Educationis one of the most influential variables on the performance of neuropsychological tests. Numerousstudies have shown that the higher the education, the better the performance.^2^ ^,^ ^3^ The moreyears of schooling, the better the performance onintelligence quotient, mathematics, visual perception, semantic and phonological processing, reasoning, and memory.^4^ ^,^ ^5^ ^,^ ^6^ ^,^ ^7^ School experienceimproves cognitive abilities as a result of specific training (e.g., asking students to frequently memorize information or solve complex computations). Moreover, attending school helps in the development of cognitive strategies that are appropriate for solving neuropsychological tests, which have a very similar format to school tests.^1^

The most common index of educational level (EL)is the number of years that the participant has attended school. Although this has been a useful index, a growing number of research studies are demonstrating that it is insufficient because it does not reflect the quality of education^8^ ^,^ ^9^ ^,^ ^10^ In this context, EL refers to the level of cognitive development attained as a consequence of the education received, whereas the quality of education refers to educational practices and resources that better improve the cognitive development of the student.It is a fact that the quality of education varies across different educational settings. There are differences between public and private schools,and between schools from different regions, different states, and different countries.^11^ ^,^ ^12^ Thus, two participants with the same number of years of schooling may have received a very different quality of education. This, in turn, may exert a considerably different influence on the cognitive performance of each one.

Some researchers have proposed an alternative approach to this problem. They advocated the use of qualitative measures of EL. These qualitative measures mainly involve reading. As reading is a skill generally acquired at school and is directly associated with the quality of education received, it is considered an accurate indicator of EL.^10^ Although reading performance is strongly correlated with EL, the latter involves other elements beyond reading such as crystallized knowledge, vocabulary, or cognitive strategies among others. The use of reading tasks as an intervening variable has helped explain differences in the cognitive performance of racial groups. For example, Manly etal. found that after adjusting the scores for a reading score, differences between African Americans and Whites in a battery of neuropsychological tests were greatly reduced for most of the tests.^9^

Overall, researchers have used the reading of irregular words as a proxy for EL.^8^ ^,^ ^9^ However, this approach seems appropriate for highly irregular or opaque languages such as English or French. For more regular or transparent languages, such as Spanish, Finnish, or Italian, this approach might be quite difficult to apply. Therefore, an alternative approach to measure EL is to assess RF. RF is “the oral translation of texts with speed and accuracy”.^13^ Several studies have found that RF increases as the number of years of schooling increases.^14^ ^,^ ^15^ ^,^ ^16^ Andrade, Celeste, and Alves found that the mean of correct words per minute rose from 137.7 in sixth grade to 149.9 in ninth grade in children from public schools.^14^ Biemiller using the years from second grade to adulthood found similar results. In his sample, when participants were given a text to read, mean time in seconds exhibited a decrease from 0.53 in second grade to 0.18 in adults.^15^ Thus, research has shown that increased number of years spent in school is related to a better RF. RF is also related to reading comprehension.^13^ Above all, learning and school performance are strongly related to RF.^17^ ^,^ ^18^ Bigozzi et al. found significant correlations between RF and school performance in grades 4 through 9.^17^ Stage and Jacobsen found significant correlations between RF and scores on a standard school achievement test at three different points in time in a group of fourth graders.^18^ Thus, RF can be considered a good indicator of the EL.

At present, there are no studies relating RF and neuropsychological test performance. The objective of this study is to compare the relationship between two indexes of EL, RF, and number of years of education, with the performance on the Multicultural Neuropsychological Scale (MUNS) in a control sample. It is hypothesized that RF will present a stronger correlation to cognitive performance than the number of years of schooling.

## METHODS

### Sample

All participants gave their informed consent to participate in this study. Participants were not included in the analysis if they had any history of neurological disease, psychiatric diagnosis,diabetes, head trauma, heart attack, non-controlled high blood pressure, coma, drug intake, alcoholism, sleep disorders, uncorrected visual impairment, communication disorders, learning disabilities, or chronic headaches. The data are obtained through a detailed questionnaire administered to each participant. The questionnaire used was thorough and has proven to be accurate to exclude patients with conditions that might affect brain functioning in the previous studies.^19^ The final sample consisted of 56 unpaid volunteers as 15 participants were excluded from the original sample of 71 participants. They were recruited from both urban and rural areas of the province of Córdoba in Argentina with a convenience sample. Participants were recruited from several sources including individuals attending teaching programs for older adults as well as acquaintances or relatives of the test administrators. Seventy percent of the participants were females. The mean age was 35.9±20 (range: 17–87 years). Table 1 shows the demographic characteristics of the sample.

**Table 1.** Demographic characteristics of the sample (n=56).

Demographic variables	Descriptive statistics
Age, mean(SD)	36(20); range (18-87)
Years of school, mean (SD)	8.7 (3.7); range (3-19)
Male, n (%)	17 (30%)
Female, n (%)	39 (70%)

### Instruments

Reading fluency task: This task consisted of a text that described the weather of a city (Córdoba, Argentina). The text, in Spanish, was 215 words, separated into 5 paragraphs. It was extracted from a free content web page and was modified in order to achieve a neutral emotional tone. The text was presented in 12-point “Times New Roman” font on an A4 size sheet. Participants were asked to place the text at a comfortable reading distance and read it aloud at their usual reading pace. The reading performance was audio recorded to accurately score for errors and reading time. Omissions, substitutions, insertions, and self-corrections were deemed as errors. The score was the number of words read correctly per minute. The following formula was used to obtain the score: (60 × (215 - errors))/total time in seconds.

MUNS:^20^this scale is a screening tool that includes seven subtests measuring five domains, namely, attention, memory, language, executive functions, and constructional praxis (see Fernández et al. for a detailed description).^20^ The total score of the MUNS showed a normal distribution. Preliminary results on validity and reliability showed a sensitivity of 86.4% and a specificity of 60.9% to discriminate normal controls from a group of cognitively impaired participants. The reliability test–retest coefficient was 0.82.^21^

Both the MUNS and the RF task were administered by properly trained psychology students in a single session.

Pearson’s *r* was used to evaluate the linear correlation between these variables that can be treated as interval variables. The Student’s *t*-test was used because of the small sample size.

## RESULTS

**Figure 1.**
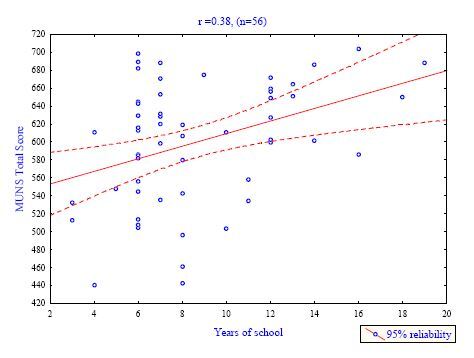
Correlation between MUNS total score and years of school.

**Figure 2.**
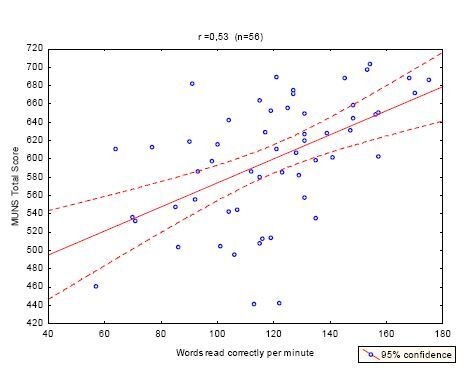
Correlation between MUNS total score and words read correctly per minute.

Table 3 shows the correlations between the MUNS scores, RF, and years in school. Figures 1 and 2 show these correlations. Age correlated significantly with the MUNS total score (*r*=-0.34; p<0.05). Partial correlations controlling for the age were obtained, showing a small effect of age as an intervening variable. Years of schooling correlated 0.34; p=0.012, whereas RF correlated 0.51; p=0.000. When only the age range of 18–60 years was considered, MUNS scores correlated 0.53 with words read correctly per minute and 0.31 with years of schooling. There were no differences in the performance between males and females on the MUNS total score Student’s *t*-test (54) −1.03, p=0.3.

**Table 3.** Correlations between MUNS total score and years of school and words read correctly per minute.

	Years of school	Words read correctly per minute
MUNS total score	r=0.38, p<0.004	r=0.53, p<0.000

MUNS: Multicultural Neuropsychological Scale.

## DISCUSSION

In this study, the score of an RF task had a stronger correlation than years of schooling with the scores of a neuropsychological scale. This stronger correlation suggests that RF might be a more accurate indicator of EL than years of schooling. As observed in Figure 1, participants with the same number of years of schooling showed a disparate performance on the neuropsychological scale. For example, for those with 6 years of schooling, the score range extended from 500 to 700 points. This is especially noticeable among those participants with 6–8 and 12 years of schooling. Interestingly, these periods match the end of primary school (6 or 7 years according to different provinces) and secondary school (12 years) in Argentina. This finding might reflect some disparities between schools in terms of the quality of education. Given the same number of years of schooling, students from different schools may have developed dissimilar RF and/or cognitive abilities.

This fact becomes relevant when education quality is considered. In poor educational systems, some students may have completed the basic education phase. However, the level of the abilities they have developed might be well below other students enrolled in higher quality systems. In fact, the Program for International Student Assessment (PISA) evaluations demonstrate that students from different countries in the world show very disparate performances on tasks measuring basic school abilities, such as reading and mathematics, even when they have completed the same number of years of schooling.^22^

It seems appropriate to consider that RF is an indirect measure of EL. Its relationship is most likely mediated by intervening variables such as working memory and reading comprehension. Different studies have shown that RF correlates positively with reading comprehension.^23^ ^,^ ^24^ Since reading demands holding information in mind, one of the crucial cognitive functions in RF performance is working memory. Therefore, a more automatic reading demands less working memory capacity for decoding which means that these resources can then be applied to comprehension.^25^ Better reading comprehension, in turn, influences school outcomes.^13^ ^,^ ^17^ Baştuğ, for example, used structural equation modeling in a sample of 1,028 participants and demonstrated that reading comprehension positively predicted academic outcomes.^26^ In addition, Bigozzi et al. using regression analyses found that RF significantly predicted school marks (i.e., Italian, English, History, Geography, Mathematics, Sciences, Technology, Music, Art, and Physical Education) in all participants.^17^ Guldenoglu also affirmed that reading comprehension influences academic outcomes and is a prerequisite for various academic skills.^27^

Using RF as a measure of EL may be a better indicator than reading irregular words as it might be applied to many languages. The Early Grade Reading Assessment (EGRA) is an example of the versatility of an RF task as a multilingual test. The EGRA is one tool used to measure students’ progress in learning to read. One of its subtests is the Oral Reading Fluency with Comprehension. This 1-min RF subtest has been applied in 70 languages, and it is used to estimate the reading comprehension abilities of children.^28^ Thus, a specific text used for an RF test can be translated into multiple languages, which facilitates cross-cultural applications and comparisons, whereas using irregular words implicates the more arduous task of selecting the appropriate words for each language. Although mean RF scores for each language will vary, the stimuli are constant. Moreover, another study demonstrated that the brain regions and cognitive abilities involved in reading are the same across different languages, either if their writing systems are alphabetic or logographic.^29^

The use of RF as a measure of EL is also convenient in terms of the use of time. In this study, it was possible to estimate the RF with a test that, on average, required 2 min to administer (Table 2).

**Table 2.** Means and standard deviations of the variables under analysis.

	n	Mean	Range	SD
Years of school	56	8.7	3-19	3.7
Words read correctly per minute	56	119.9	57-175	27.3
MUNS total score	56	599.9	441-704	67.7
Time to read the passage	56	110.6	71-203	29.4

Words Read Correctly per Minute were taken from the reading fluency task. SD: standard deviation; MUNS: Multicultural Neuropsychological Scale.

One limitation of this study is the small sample size. This may have had an influence on the magnitude of the correlations. Future studies should replicate these results with larger samples. In addition, the influence of age, although small, may have introduced some confounding factors. It is possible that some elderly participants had a decreased RF as a result of a processing speed decrease considering its significant participation in the performance of the RF test and not as a reflection of a lower EL.^30^ Furthermore, the inclusion criteria might have produced some false negatives. However, the normal distribution of the MUNS scores presents some evidence that very few or none of the false negatives was included.^20^

In sum, RF can be considered a proxy for EL in future studies involving neuropsychological test performance.
